# Non-aneurysmal subarachnoid hemorrhage in a 13-year-old male with infective endocarditis caused by *Abiotrophia defectiva*: a case report and literature review

**DOI:** 10.3389/fped.2025.1625229

**Published:** 2025-07-22

**Authors:** Anas K. Assi, Habeeb H. Awwad, Riman A. Sultan, Zaina A. Khaled, Sara N. Fatoum, Abdalwahab Kharousha, Hadi Dababseh, Rabee Adwan

**Affiliations:** ^1^ Faculty of Medicine, Al-Quds University, Jerusalem, Palestine; ^2^ Department of Neurosurgery, Istishari Arab Hospital, Ramallah, Palestine; ^3^ Department of Infectious Diseases, Istishari Arab Hospital, Ramallah, Palestine

**Keywords:** infective endocarditis, *A. defectiva*, subarachnoid hemorrhage, mitral valve prolapse, pediatric neurology

## Abstract

**Introduction:**

Infective endocarditis (IE) is a rare but serious condition. It is commonly caused by *viridans group streptococci* (VGS) or *Staphylococcus aureus*, often in the presence of structural heart disease. Rare organisms, like *Abiotrophia defective* (A. defective), can cause IE. Although resistance may not be present in every case, infections caused by this organism are often associated with a more complicated clinical course and may require tailored treatment strategies. In this case, antimicrobial susceptibility testing indicated broad susceptibility, and no resistance was observed in the isolated strain.

**Case presentation:**

A 13-year-old boy arrived with a severe headache and fever. He had previously been misdiagnosed with infective endocarditis (IE) caused by VGS based on blood cultures and echocardiography, which revealed severe mitral regurgitation (MR), and he received treatment. Despite therapy, he developed a non-aneurysmal subarachnoid hemorrhage (SAH), as confirmed by CT and MRI. A repeat echocardiography revealed mitral valve prolapse (MVP), new vegetations, and pericardial effusion, as a complication of A. defectiva identified later on blood cultures as the causative organism, which led to a revised diagnosis of IE*.* He remained stable with appropriate treatment, with no neurological abnormalities.

**Conclusion:**

We report a case of A. defectiva IE that is exacerbated by non-aneurysmal SAH. It emphasizes the significance of taking IE into account in kids who have neurological symptoms, as well as the necessity of close observation for uncommon but serious causative organisms.

## Introduction

Infective endocarditis (IE) is a rare infectious illness, with an annual incidence of 3–7 per 100,000 person-years in recent studies. Despite its rarity, IE is associated with increased morbidity and mortality, and it is currently the third most prevalent life-threatening infection syndrome, behind sepsis and pneumonia (1). Pediatric IE is frequently linked to underlying structural heart problems, such as regurgitation or mitral valve prolapse (MVP). It's usually caused by *viridans group streptococci* (VGS) or *Staphylococcus aureus*, and less frequently by organisms like *Abiotrophia defectiva* (A. defectiva) (2).

Up to 40% of patients with IE experience neurological consequences, which might include subarachnoid hemorrhage (SAH), intracerebral hemorrhage, or embolic stroke (3). Non- aneurysmal SAH is an uncommon but potentially fatal complication of IE and frequently linked to septic emboli or mycotic aneurysms (4).

A. defectiva was first reported in 1961 by Frenkel and Hirsch. It was described as a microorganism that belongs to a Nutritionally Variant Streptococci (NVS) with a high death rate and resistance to antibiotics (5, 6). In 1989, Bouvet et al. (7) separated NVS into Streptococcus defectivus and Streptococcus adjacent according to DNA homology using DNA–DNA hybridization. Later, 16S rRNA sequencing was used to move them to a new genus, Abiotrophia (8). Traditionally, A. defectiva has been defined as non-motile, pyridoxine- dependent, catalase-negative, Gram-positive cocci in chains that display satellitism (9, 10).

Five percent of all instances of IE and five to six percent of all cases of streptococcal endocarditis are caused by Abiotrophia (3). As it has been identified as part of the normal flora of the human oral cavity, gastrointestinal tract, and urogenital tract (8). A. defectiva can cause bacteremia and infective endocarditis, but it has also been implicated in other infections such as sinusitis, osteomyelitis, corneal ulcers, and scrotal abscesses. Although it's an uncommon cause of endocarditis, the clinical outcome can be severe, with the need for early surgical intervention (5).

IE produced by this microbe is more difficult to treat microbiologically than IE caused by a strain of non-nutritionally variant VGS. According to the American Heart Association guidelines for the management of infective endocarditis in children, pediatric patients with IE caused by A. defectiva should receive a combination of intravenous penicillin G (250,000–300,000 units/kg/day divided every 4–6 h; maximum 18–24 million units/day) or ampicillin (200–300 mg/kg/day divided every 4–6 h; maximum 12 g/day) plus gentamicin (3 mg/kg/day divided every 8 h) for 4–6 weeks, with all doses adjusted for weight and renal function. Ceftriaxone (100 mg/kg/day IV once daily; maximum 4 g/day) is an acceptable alternative to penicillin or ampicillin if susceptibility is confirmed. Infectious diseases specialist consultation is recommended to individualize therapy and determine optimal duration, especially given the organism's fastidious nature and the potential for complications (11).

We report a 13-year-old child with IE, initially attributed to VGS, but later identified as A. defectiva upon re-evaluation and additional testing. The condition is worsened by severe mitral regurgitation and a spontaneous non-aneurysmal subarachnoid hemorrhage. This case demonstrates the difficulties in diagnosing and treating pediatric IE, especially when uncommon infections and neurological aftereffects are involved.

## Case presentation

A 13-year-old boy was admitted to our neurosurgical department due to a severe headache and fever. Three months prior to admission, he developed a severe frontal headache that awakened him from sleep with no response to analgesia, followed by a seizure and loss of consciousness. Accordingly, a non-contrast computed tomography (CT) scan was done and revealed no abnormality, he was admitted and managed in the intensive care unit (ICU) for 24 h and then discharged on phenytoin (100 mg twice daily). Subsequently, the patient developed a high-grade fever and presented to an outpatient clinic, where he was prescribed a 10-day course of Amoxicillin/clavulanic acid (875 mg–125 mg twice daily). Although initial symptoms subsided, the fever recurred shortly after completing the first course, prompting a second consultation at the same clinic and administration of an additional course of Amoxicillin/clavulanic acid.

Despite that, he continued to experience recurrent febrile episodes and persistent headaches, significantly impacting his daily functioning. Due to persistent symptom, the patient came to the hospital for further evaluation and labs revealed elevated inflammatory markers, including erythrocyte sedimentation rate (ESR), C-reactive protein (CRP), and a positive rheumatoid factor (RF).

A transthoracic echocardiogram (TTE) was done and showed severe mitral regurgitation (MR) without vegetations. A total of two sets of blood cultures were obtained, in which they grew VGS. Ultimately, the patient was diagnosed with infective endocarditis (IE) meeting the modified Duke's criteria: one major criteria (positive blood cultures for a typical IE organism) and three minor criteria (fever, predisposing heart abnormality, positive rheumatoid factor). Antibiotic susceptibility testing was conducted and sensitive to cefotaxime, vancomycin, ceftriaxone and ampicillin. He was initiated on a combination of gentamicin (80 mg twice daily) and ceftriaxone (2 g twice daily) for 2 weeks. Before discharge, a transesophageal echocardiogram (TEE) confirmed the presence of severe MR and mitral valve prolapse (MVP) with no vegetation, and his symptoms resolved. He was discharged ceftriaxone (2 g twice daily) for another 2 weeks. Following discharge, he developed Osler nodes and conjunctivitis. A follow-up echocardiogram revealed no increase in the severity of regurgitation and no evidence of vegetations. At the time of initial presentation, the source of the infective endocarditis was unclear, with no identifiable focus of infection.

**Figure 1:**
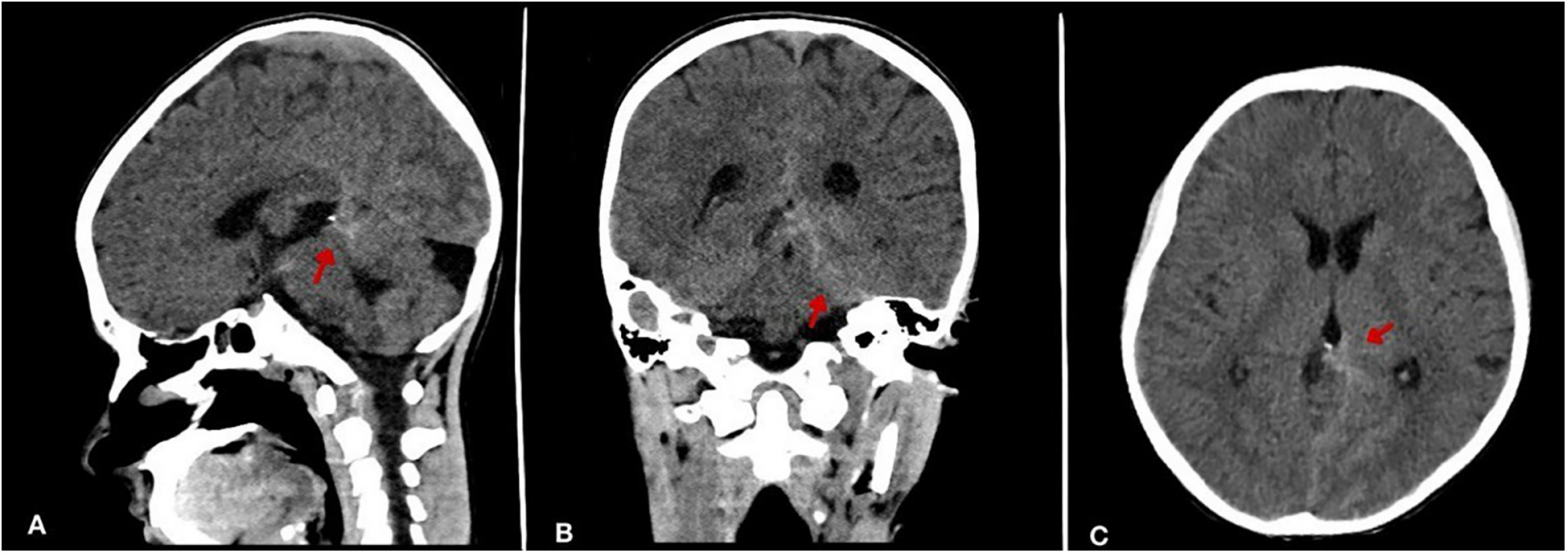
Non-contrast brain CT demonstrating subarachnoid hemorrhage (SAH). **(A)** Sagittal view shows hyperdensity (arrow) within the interhemispheric fissure and basal cisterns. **(B)** Coronal view reveals prominent hemorrhage (arrow) in perimesencephalic cisterns. **(C)** Axial section confirms diffuse hyperdensity (arrow) in basal cisterns.

The patient experienced multiple episodes of high grade fever since then. However, he was stable until one day before admission, when he developed a severe frontal headache and fever of 38.7°C. Therefore, a non-contrast computed tomography (CT) scan was done and revealed a left peri-mesencephalic subarachnoid hemorrhage (SAH) and a contrasted CT showed no evidence of aneurysmal malformation (Figure 1). As a result, he was referred to our hospital for neurosurgical evaluation and ICU management. His past medical and surgical history was otherwise unremarkable, with no history of immunosuppressive therapy, known allergies or inherited diseases. He denied any recent dental or invasive procedure.

On physical examination, the patient was conscious, alert, hemodynamically stable, and afebrile. Neurological assessment showed an alert patient with a Glasgow Coma Scale score of 15.

Muscle strength and tone were normal, with negative meningeal signs, intact cranial nerves, and no evidence of motor deficits, or seizures. Cardiac examination revealed an apical pan-systolic murmur. Laboratory tests showed a normal leukocyte of 10.92 × 10³/μl and platelet count 375 × 10³/μl, a low hemoglobin level of 8.4 g/dl, an elevated CRP of 35 mg/L, and normal creatinine levels. Troponin levels were within normal range.

**Figure 2:**
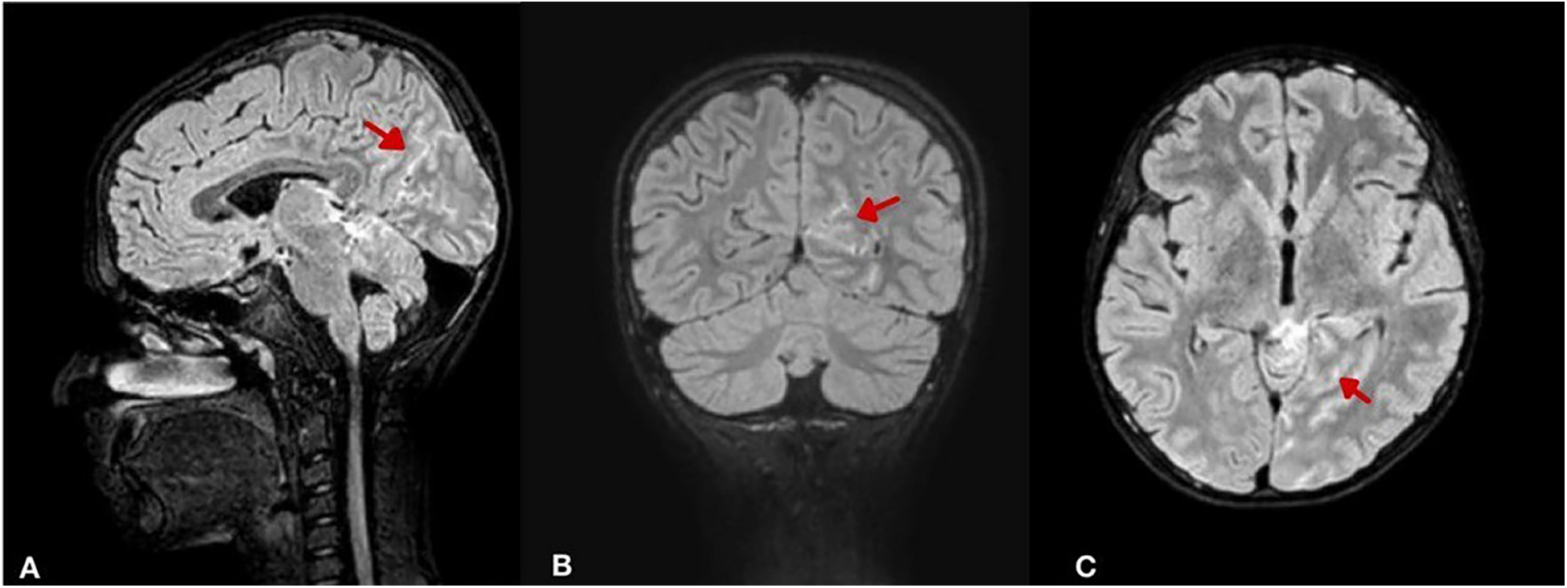
Brain MRI (FLAIR sequence with intravenous contrast) illustrating SAH distribution and secondary changes. **(A)** Sagittal view shows hyperintense signal (arrow) in the perimesencephalic, ambient, and quadrigeminal cisterns. **(B)** Coronal view reveals left occipito-parietal cortical hyperintensities (arrow) suggesting cortical involvement. **(C)** Axial view shows a focal right cerebellar hyperintensity (arrow) with bilateral periventricular and sulcal hyperintensities, indicating possible ischemic sequelae.

An urgent magnetic resonance imaging (MRI) scan confirmed the presence of a subarachnoid hemorrhage, primarily around the midbrain and basal cisterns, particularly in the left prepontine, ambient, and quadrigeminal cisterns, as well as in the left occipito-parietal lobes. Additionally, a small focus of diffusion restriction with high T2-weighted/FLAIR signal intensity and low T1- weighted signal was observed in the right cerebellar hemisphere. Bilateral periventricular foci of high FLAIR signal and scattered tiny foci of high diffusion-weighted imaging (DWI) signal were also noted, likely reflecting small areas of diffusion restriction and possible sequelae of SAH (Figure 2). A follow-up CT scan showed no significant changes and the SAH was managed conservatively.

A repeat echocardiogram demonstrated MVP with moderate MR and an echogenic lesion on the atrial surface of the anterior mitral leaflet, most likely representing vegetations. A small pericardial effusion was also noted (ECHO).A total of three sets of blood cultures were taken within 24 h of admission, each using a pediatric blood culture bottle with a minimum of 3 ml blood. A repeat blood cultures were obtained, and empiric antibiotic therapy with ampicillin (2 g every 4 h) and gentamicin (80 mg every 12 h) was initiated. Culture results confirmed the presence of a Gram-positive cocci and finally was identified as A. defectiva sensitive to teicoplanin, linezolid, cefotaxime, vancomycin, ceftriaxone and ampicillin. The identification and drug sensitivity testing were performed using the VITEK 2 Compact system. Antibiotics were switched to ceftriaxone (2 g morning, 1.5 g at night).

The patient remained stable, tolerating oral feeding and off intravenous fluids. He was discharged on day 12 of admission with improved symptoms and a normal neurological examination. He was prescribed daily ceftriaxone (2 g morning, 1.5 g at night) for 4 weeks and levetiracetam (500 mg twice daily) for 1 year. All dosing was weight-based, consistent with pediatric recommendations. The patient was managed in consultation with pediatric infectious diseases specialist. And was scheduled for follow-up with the pediatric neurosurgery and neurology department in 2 weeks, as well as the cardiology clinic with a repeat echocardiogram planned 1 week after discharge.

At the most recent cardiology follow-up, the patient remained asymptomatic with no recurrence of fever, headache, or neurological deficits since discharge. Serial echocardiograms showed a sustained improvement, with the left ventricular end-diastolic diameter (LVEDd) measuring 4.8 cm, the ejection fraction (EF) preserved at 75%, and no pericardial effusion. The vegetation on the atrial surface of the anterior mitral leaflet had decreased in size from 13 mm to 6 mm. Mild MR persisted, the left atrium and ventricle remained normal in size, and there was no evidence of new valvular destruction or perivalvular complications. Given these findings and the patient's clinical stability, the multidisciplinary team opted to continue conservative management, with close surveillance through monthly cardiology evaluations.

## Discussion

A. defectiva is a facultative anaerobic Gram-positive coccus (12). It is considered part of the normal microbiota of the oral cavity, gastrointestinal tract, and genitourinary system (13). Regarding its pathogenic mechanisms, it has multiple unique methods, beginning with adherence to the endocardium by secreting exopolysaccharides, which enhance binding to fibronectin on the extracellular matrix (12). This is followed by a biofilm formation, which facilitates persistent infection and resistance to host defenses. These specialized mechanisms confer a high affinity for endovascular structures, leading to vegetation formation and valvular damage (14). A. defectiva has been found to be one of the uncommon causes of infective endocarditis in pediatrics, which typically occurs in children with congenital heart disease or a history of previous cardiac surgery, but that doesn't exclude being a cause of IE even in healthy patients with no underlying diseases (15). It also can cause serious infections such as bacteremia, brain abscess, osteomyelitis, septic arthritis, and pancreatic abscess (16).

Compared to adults, children are less likely to have IE. According to estimates, 0.43–0.69 per 100,000 children are diagnosed with IE annually (17). In the US, there are between 0.05 and 0.12 occurrences of pediatric IE hospitalization for every 1,000 pediatric admissions each year (11).

Less than 1% of all IE cases and roughly 5%–6% of all streptococcal endocarditis cases are caused by the uncommon IE cause A. defectiva (13, 16). Moreover, according to the literature, only a few pediatric cases have been reported with IE caused by A. defectiva worldwide, making it extremely rare and can lead to significant morbidity and mortality (18). While rheumatic fever and central venous catheter are known risk factors for IE in pediatrics, congenital heart disease is still the primary predisposing factor so far (18). Although our case of A. defectiva IE was in a previously healthy 13-year-old boy with no known structural heart disease or prior interventions, severe MR and MVP were discovered during the workup for IE and are likely secondary to the infectious process.

Nonspecific symptoms include new or altered heart murmur (68% of cases), splenomegaly (70%), and persistent fever (89%) are common in pediatrics IE (19). Compared to adults, children are less likely to develop immunologic phenomena like Osler nodes and Janeway lesions (19). IE can lead to multiple complications, which can be classified into two categories; cardiac and extracardiac. They are more frequent in children without known heart disease and those who are less than 2 years old. Additionally, children are less likely than adults to experience IE complications (17). Extracardiac complications include neurological consequences mainly ischemic stroke, which is the most common one and accounts for 25% of cases. With multiple other manifestations that still can occur like SAH, meningitis, seizure and brain abscess.

In our patient, the SAH was non-aneurysmal, confirmed via contrast-enhanced CT and MRI, and it was particularly obvious in the occipitoparietal and left perimesencephalic areas. This finding is highly atypical in pediatric IE, where SAH, when it occurs, is more commonly aneurysmal (4, 16). In contrast to the majority of reported cases where mycotic aneurysms are implicated, imaging in our patient revealed no vascular malformations, suggesting a different mechanism.

According to the assessment by both the radiology and neurosurgery teams, the subarachnoid hemorrhage in this case was most likely a consequence of septic emboli originating from the infective endocarditis. These emboli, typically composed of fragments of valvular vegetations, bacteria, and fibrin, can travel to the cerebral vasculature. While mycotic aneurysms are a common cause of SAH in IE, septic emboli can also lead to hemorrhage through other mechanisms, such as septic vasculitis or microvascular rupture due to direct bacterial invasion of the vessel walls, even without macro-aneurysm formation (1, 12). The patient's clinical improvement following targeted antibiotic therapy further supports the embolic and infectious nature of the neurological complication, as bacterial load reduction would mitigate ongoing embolic events and inflammatory damage to vessels. Given the likely septic embolic etiology, thromboprophylaxis was not indicated, and anticoagulation was carefully avoided due to the inherent increased risk of exacerbating intracranial bleeding in the context of active hemorrhage.

Overall, A. defectiva IE is extremely challenging to diagnose. Due to its subacute clinical cause, fastidious growth requirements, and frequent presentation as culture-negative endocarditis, many A. defectiva infections may be misdiagnosed with other pathogens, such as VGS (16). According to estimates, up to half of endocarditis with negative cultures may be caused by nutritionally variant streptococcus (NVS) as A. defectiva (14). Diagnostic techniques, including positive blood cultures, vegetations on echocardiography, neuroimaging, and cerebral angiography, are required for diagnosis and detecting SAH in such A. defectiva- caused IE cases. Notably, the diagnosis in our case evolved from an initial identification of VGS diagnosis to confirmed A. defectiva IE upon readmission, highlighting the pathogen's ability to masquerade as more common organisms and the importance of repeat blood cultures and echocardiography in cases with atypical or relapsing courses.

The management was greatly impacted by identification of A. defectiva. It usually involves long-term antibiotics, primarily beta lactams as penicillin or ceftriaxone in combination with gentamicin (13). But, following sensitivity testing, ceftriaxone, a third-generation cephalosporin was chosen for prompt treatment due to the organism's known resistance profile. This is consistent with the most recent recommendations. However, valve surgery may be required for large vegetation (>10 mm), recurrent embolism, or heart failure due to valvular dysfunction (17).

In case of neurological complications such as SAH, immediate stabilization and blood pressure control are critical. Neurosurgical intervention may be needed for ruptured aneurysms, and anticoagulation should generally be avoided initially due to bleeding risk.

The mortality rate for pediatric IE varies depending on the causative organism and complications (12). However, A. defectiva has a higher mortality rate compared to other streptococci, reaching up to 17%, with a higher relapsing rate despite appropriate antibiotic therapy (14). Patients with A. defectiva endocarditis compared to VGS have higher rates of periannular complications (28.9% vs. 22%). Cardiac surgical intervention is also more frequent (65.8% vs. 50%) (17). In our case, despite the presence of mitral regurgitation and vegetations, valve surgery was avoided, unlike many cases in which surgical intervention is required due to poor response or structural damage. This favorable outcome is especially noteworthy given the high relapse and complication rates associated with A. defectiva.

This case of pediatric infective endocarditis (IE) caused by A. defectiva exhibits several unique features compared to the limited existing literature. While A. defectiva accounts for less than 1% of all IE cases globally (12), its occurrence in children is exceptionally rare, with only a few reported pediatric cases worldwide prior to this report (20). Unlike most pediatric IE cases linked to congenital heart disease or prior cardiac surgery, this patient had no structural cardiac abnormalities before developing mitral valve prolapse and severe regurgitation during the infection. The co-occurrence of non-aneurysmal SAH further distinguishes this case, as SAH in IE typically arises from mycotic aneurysms. Additionally, despite A. defectiva association with higher mortality and relapse rates compared to other streptococci, this patient achieved stabilization with ceftriaxone monotherapy after initial combination antibiotics, avoiding valve surgery often required in refractory cases. The absence of congenital risk factors and the successful medical management of both IE and SAH make this case a valuable addition to the sparse pediatric literature on A. defectiva infections.

## Conclusion

This case describes an uncommon form of pediatric IE accompanied by non-aneurysmal SAH and infection with A. defectiva. It emphasizes the necessity of investigating IE in children with unexplained fever and neurological symptoms, as well as the need for close monitoring for uncommon but serious consequences such as cerebral bleeding. Early recognition, appropriate antibiotic therapy, and close multidisciplinary follow-up are critical to improving outcomes in such complex cases.

## Data Availability

The original contributions presented in the study are included in the article/Supplementary Material, further inquiries can be directed to the corresponding author.
